# *Nitzschia anatoliensis* sp. nov., a cryptic diatom species from the highly alkaline Van Lake (Turkey)

**DOI:** 10.7717/peerj.12220

**Published:** 2021-10-22

**Authors:** Cüneyt Nadir Solak, Romain Gastineau, Claude Lemieux, Monique Turmel, Ewa Gorecka, Rosa Trobajo, Mateusz Rybak, Elif Yılmaz, Andrzej Witkowski

**Affiliations:** 1 Department of Biology, Arts and Science Faculty, Dumlupınar University, Kütahya, Turkey; 2 Institute of Marine and Environmental Sciences, University of Szczecin, Szczecin, Poland; 3 Département de biochimie, de microbiologie et de bio-informatique, Institut de Biologie Intégrative et des Systèmes, Université Laval, Québec, Québec, Canada; 4 Marine and Continental Waters Program, IRTA-Institute of Agriculture and Food Research and Technology, Sant Carles de la Ràpita, Catalonia, Spain; 5 Department of Agroecology and Forest Utilization, Institute of Agricultural Sciences, Land Management and Environmental Protection, University of Rzeszów, Rzeszów, Poland

**Keywords:** Diatoms, New species, Extreme habitats, Van Lake, Alkaline lake, Cryptic diversity, Organellar genomes, Multigene phylogeny

## Abstract

In this article we describe *Nitzschia anatoliensis Górecka, Gastineau & Solak* sp. nov., an example of a diatom species inhabiting extreme habitats. The new species has been isolated and successfully grown from the highly alkaline Van Lake in East Turkey. The description is based on morphology (light and scanning electron microscopy), the sequencing of its organellar genomes and several molecular phylogenies. This species could easily be overlooked because of its extreme similarity to *Nitzschia aurariae* but molecular phylogenies indicate that they are only distantly related. Furthermore, molecular data suggest that *N. anatoliensis* may occur in several alkaline lakes of Asia Minor and Siberia, but was previously misidentified as *Nitzschia communis*. It also revealed the very close genetic proximity between *N. anatoliensis* and the endosymbiont of the dinotom *Kryptoperidinium foliaceum*, providing additional clues on what might have been the original species of diatoms to enter symbiosis.

## Introduction

*Nitzschia* A.H. Hassall 1845 is the most speciose genus within the diatom family Bacillariaceae Ehrenberg and is regarded as one of the most speciose among diatoms in general. The two largest data bases on taxonomy and species richness, WORMS and Algaebase, provide a number of species for *Nitzschia* well exceeding 1,000. WORMS lists 1,495 taxa (Kociolek et al., 2018), while Algaebase lists 1,284 species and 442 infraspecific names (Guiry & Guiry, 2019), with 842 flagged as accepted taxonomically. Although it is difficult to standardize *Nitzschia* morphology in terms of valve outline, numerous species represent one of the following shapes: (a) narrow, straight or narrow sigmoid; (b) narrow linear; (c) lanceolate or (d) elliptic, with usually uniseriate striae (Mann, 1978; Krammer & Lange-Bertalot, 1988; Round, Crawford & Mann, 1990). The raphe system in *Nitzschia* is either slightly (sometimes close to central) to strongly eccentric, almost marginal (Mann, 1978; Krammer & Lange-Bertalot, 1988; Round, Crawford & Mann, 1990).

Numerous taxa belonging to *Nitzschia* are of great importance for hydrobiologists, ecologists and water quality assessment specialists, as they have very narrow environmental tolerance and are readily applied for water quality monitoring (Alakananda et al., 2011; Rimet, 2011; Solak & Ács, 2011; Trobajo et al., 2013). However, other *Nitzschia* species are very resistant and can tolerate high concentrations of lethal compounds, including organic pollutants and the most degraded industrial and municipal waters (Bates et al., 2018). Although most *Nitzschia* species inhabit benthic habitats (Round, Crawford & Mann, 1990), numerous ones are major components of plankton communities, especially the species found in large lakes, for instance the Great lakes of the East African rift zone (*e.g*. Sarmento, Isumbisho & Descy, 2006; Stager et al., 2009). Therefore, enhanced knowledge on autecological characteristics of *Nitzschia* species proved useful not only for biomonitoring programs but also for environmental reconstructions (*e.g*. Horton, Boreham & Hillier, 2006; Beyene et al., 2009; Trobajo et al., 2013).

Among the extreme habitats hosting diatoms are saline lakes and alkaline lakes. However, these environments and their diatoms are understudied compared to freshwaters and may reveal unexpected and cryptic biodiversity. For example, a new species of *Nitzschia*, whose abundance was linked with the degradation of wetlands, was discovered in Central European alkaline saline lakes (Földi et al., 2018). The Great Salt Lake in Utah, another inland alkaline lake, is known for hosting several species of *Nitzschia* spp. (Patrick, 1936). Other examples of such extreme environments are some African crater lakes (also with high pH), whose sediments have proven to be very rich in several *Nitzschia* species, including a very abundant new species, *Nitzschia fenestralis* (Grady, Mann & Trobajo, 2020).

Turkey is another region rich in soda lakes, the most renowned being Salda Lake and Van Lake (respectively known as Salda Gölü and Van Gölü in Turkish). Van Lake, which is also the largest lake in Turkey, is located at a high altitude (1,648 m a.s.l.) in Eastern Anatolia. It is 450-m deep with 576 km^3^ of volume, thus the largest soda lake and third largest closed lake in the World. The characteristics in terms of hydrology and water chemistry of Van Lake and the rivers draining into it have been detailed by Reimer (1995) and Reimer, Landmann & Kempe (2009). This saline lake is defined by sodium and potassium, balance of bicarbonate and carbonate ions with alkaline earth ions, a Na-CO_3_-Cl-(SO_4_)-chemistry (Reimer, Landmann & Kempe, 2009), a conductivity of 22.9–26.7 mS.cm^−1^ and a pH of 9.31–9.88. The presence of diatoms in the deposits, which was first overlooked (Reimer, Landmann & Kempe, 2009), was later studied (North et al., 2018), and Van Lake is also famous for a special type of sediments called the microbialities (Kempe et al., 1991; Kempe & Kaźmierczak, 2003; López-García et al., 2005). Unique in regards to these geochemical characteristics, Van Lake also hosts endemic species such as the pearl mullet *Alburnus tarichi* Guldenstaedtii, 1814.

About 80 years ago, Legler & Krasske (1940) described several diatom species from Van Lake, some of them later reinvestigated and imaged by Lange-Bertalot et al. (1996). Among them, Legler & Krasske (1940) described a new species of *Nitzschia*, *N. incognita* and also identified several more, including *N. vitrea* G. Norman, *N. frustulum* (Kützing) Grunow, *N. inconspicua* Grunow, *N. frustulum* var. *subsalina* Hustedt, *N. fonticola* (Grunow) Grunow, *N. kuetzingiana* Hilse and *N. communis*. All of them are also listed by Gessner (1957) in his research and review of Van Lake phytoplankton and littoral diatoms species. A few reports were also published on diatoms from the surrounding area (*e.g*. Solak et al., 2012).

In the present article, we describe *Nitzschia anatoliensis* sp. nov., a new taxon isolated from Van Lake. The valve ultrastructure was characterized by means of light and scanning electron microscopy. In the frame of the current effort of genomic characterization of populations and species of diatoms (see Prasetiya et al., 2019; Gastineau et al., 2021a, 2021b), the complete organellar genomes of *N. anatoliensis* were sequenced, they were used for molecular phylogenies and compared with organellar genomes from related species.

## Material & Methods

### Sampling, isolation and cultivation

Epilithic samples were collected on May 2015 from the littoral zone of Van Lake by brushing submerged stones. Single cell was isolated using micropipettes, with further cleaning of contamination and re-inoculation until a monoclonal culture was established. The strain is now registered in the Szczecin Diatom Culture Collection as SZCZ E372. It was cultivated in 250 mL Erlenmeyer flasks with F/2 medium (Guillard, 1975) adjusted to a salinity of 20 PSU. For the light conditions, the photoperiod was 14 h light/10 h darkness with light intensity of ca. 80 µmol photons m^−2^ s^−1^ provided by fluorescent tubes.

### Microscopy

Pellets of cells obtained from the monoclonal culture were boiled with H_2_O_2_ and HCl to remove the organic matter and calcium carbonate (Renberg, 1990). After repeated washings with distilled water, the material was air-dried on cover glasses and mounted in Naphrax. Frustules were investigated under a Zeiss Axio Imager A2 light microscope (LM) equipped with a 100 × Plan Apochromatic objective with differential interference contrast (DIC) for oil immersion (NA 1.46). The images were captured with a Zeiss AxioCam ICc5 camera. Scanning electron microscope (SEM) observations were made using a Hitachi SU 8010 at the Podkarpackie Innovative Research Center of the Environment (PIRCE) at the University of Rzeszów. For this purpose, samples were dropped onto a polycarbonate membrane filter with a 3-μm mesh size, attached to aluminum stubs and sputtered coated with 20 nm of gold using a Turbo-Pumped Sputter Coater Quorum Q 150OT ES. Measurements were done using the ImageJ software (Schneider, Rasband & Eliceiri, 2012).

### Next generation sequencing and phylogenetic analysis

Cells from culture in exponential growth phase were harvested by gentle centrifugation at 900g. DNA was extracted following the protocol of Doyle & Doyle (1990). Total DNA was sequenced at the Beijing Genomic Institute (Shenzhen, China), on a BGISEQ-500. About 60 millions of 100-bp reads were produced. They were assembled with SPAdes 3.12.0 (Bankevich et al., 2012), using a k-mer value of 85. Contigs corresponding to nuclear ribosomal genes and the plastid and mitochondrial genomes were identified by customized blast analyses. Organellar genomes were completed and verified using the CONSED package (Gordon & Green, 2013) and their encoded genes were identified using the findORF tool (Gagnon, 2004). Annotation was performed using Sequin 15.50. Genome maps were generated with OGDRAW (Lohse et al., 2013). Full genome alignments were performed with progressiveMauve (Darling, Mau & Perna, 2010), with sequences from available *Nitzschia* spp. and dinotoms. For the case of the plastid genomes, the second copy of the inverted repeat was removed before alignment.

For phylogenetic inference, four different sets of genes were used: the individual nuclear small subunit (*SSU*, *18S*) and large subunit (*LSU*, *28S*) rRNA genes, the partial *rbcL* gene, 36 concatenated mitochondrial protein-coding genes, and 129 concatenated plastid protein-coding genes. Gene sequences were aligned using MAFFT 7 with the—auto option (Katoh & Standley, 2013) and variable regions were removed with trimAl with the—automated1 option (Capella-Gutiérrez, Silla-Martínez & Gabaldón, 2009). Maximum Likelihood (ML) phylogenies were inferred with RAxML version 8.0 (Stamatakis, 2014), using the GTR+I+G model. For the SSU and LSU rRNA phylogenies, a 16-state model was used to accommodate the secondary structure obtained from the RNAalifold Web Server (http://rna.tbi.univie.ac.at/cgi-bin/RNAWebSuite/RNAalifold.cgi) and the best tree out of 100 was computed for 100 bootstrap replications. For the *rbcL* phylogeny and the multigene phylogeny the best trees out of 100 were computed for 1,000 bootstrap replications.

## Results

**Figure 1:**
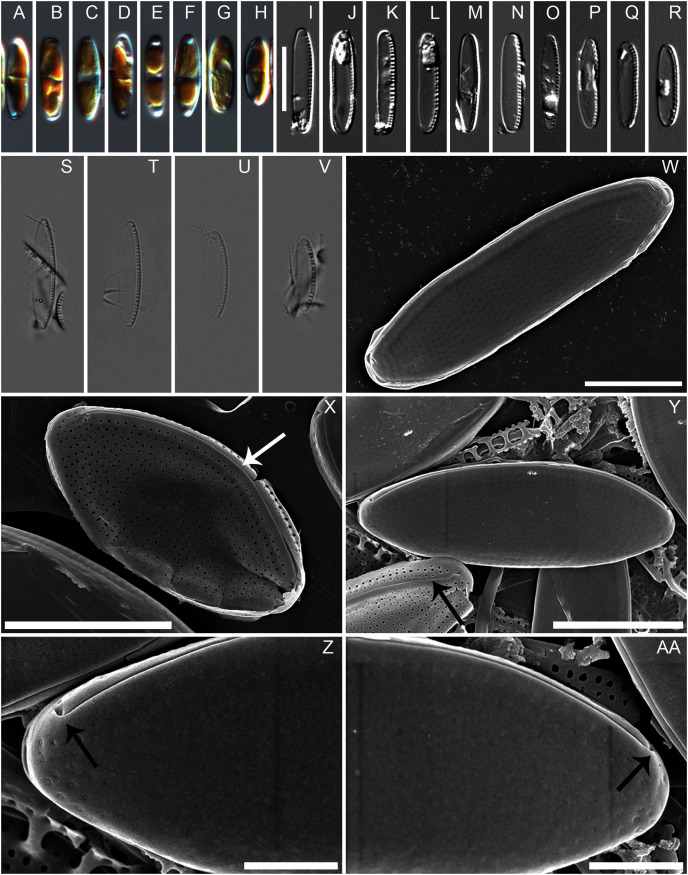
*Nitzschia anatoliensis* sp. nov. in LM and SEM. (A–H) LM images of living specimens from the culture. (I–R) LM images of cleaned valves of specimens from the culture isolated from Lake Van. (S–V) LM images of cleaned valves of specimens from wild sample. Scale bar given in I is 10 µm. (W–AA) SEM images of specimens from the culture. (W) Complete specimen, external view with the position of the canal raphe and non corroded areolae occlusions. (X) Complete specimen with corroded areolae occlusions, white arrow indicates the lack of central nodule. (Y) Complete specimen (up) and non complete (down) with corroded areolae occlusions. The black arrow indicates the row of areolae on the canal raphe and three rows of areolae on the distal valve mantle. (Z–AA): the strongly hooked apical raphe endings (arrows). Scale bars are three µm (W), three µm (X), three µm (Y), one µm (Z–AA).

***Nitzschia anatoliensis* Górecka, Gastineau & Solak sp. nov. (Fig 1–31)**

Reported as *N. communis sensu* Samylina et al. (2014); *N*. *communis sensu* Sapozhnikov et al. (2016)

**Figure 2:**
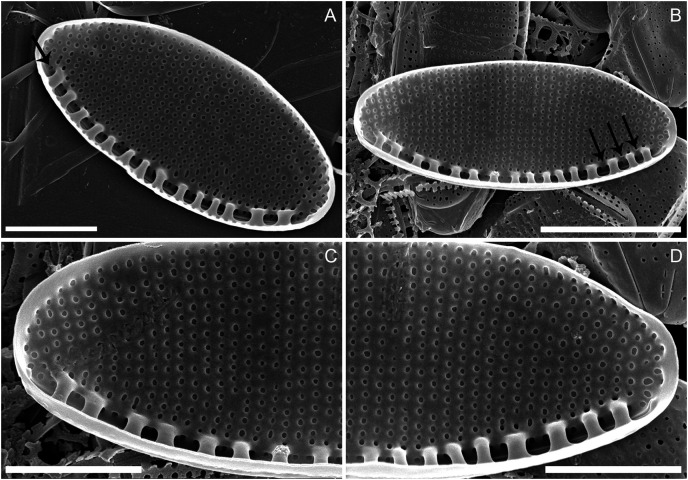
*Nitzschia anatoliensis* sp. nov. in SEM, internal view. (A) Specimen illustrating the raphe slit internally, note the absence of a break (=central nodule) and the presence of the single row of areolae on the canal raphe (arrow). (B) Complete specimen in internal view, note the presence of the small fibulae and the solitary row of areolae on the canal raphe, arrows point to the viminae marking the border between the valve face and the canal raphe. (C–D) Close up of the internal view of specimen illustrated in B. Scale bars are two µm (A), four µm (B), two µm (C and D).

*Diagnosis:* Cells with two chloroplasts, one located towards each valve end (Figs. 1A–1H). Valves linear-elliptic with broadly rounded ends, 7.8–16.1 µm long and 2.7–3.7 µm wide (*n* = 20 specimens). Canal raphe strongly eccentric, marginal, the fibulae irregularly spaced, 20–22 in 10 µm (Figs. 1I to 1V), central nodule not observed. Transapical striae in LM not resolvable, in SEM 48–52 in 10 µm (Figs. 1X, 2A to 2D). Specifically, the wild specimens (Figs. 1S to 1V) were 12–16 µm long and 3.5–4.0 µm wide, with 20–23 fibulae in 10 µm. All measurement data available as supporting information files: measurements_for_N_anatoliensis.xlsx.

*Holotype:* slide SZCZ E372 (metabolically inactive, preserved material from Szczecin Culture Collection), leg. Cüneyt Nadir Solak, May 2015.

*Type locality: *collected as epilithic samples Turkey, Van Lake (39° 56′ 7.992″ N; 42° 16′ 52.993″ E).

*Distribution:* Observed from the type locality and in soda lake of Kulunda Steppe in Altai Region of the Russian Federation.

*Etymology:* The species name is derived from the word Anatolia, the geographic region in Eastern Turkey where Van Lake is located.

*Description:* External valve surface flat with slightly elevated canal raphe. Canal raphe strongly eccentric, marginal. The raphe is filiform with external proximal raphe endings (central nodule) absent and the raphe slit running through (Figs. 1W to 1Y). The apical raphe endings strongly hooked in the same direction and terminate on the valve mantle (Figs. 1Z and 1AA). Proximal valve mantle shallow with three rows of areolae (Fig. 1Y); distal valve mantle formed by narrow, hyaline stripe of silica. Transapical striae parallel in the middle, becoming slightly radiate towards the apices and finally convergent at the apices, 48–52 in 10 μm (Figs. 1W to 1AA; Figs. 2A to 2D) composed of small and oblong to circular areolae, ca. 55 in 10 μm (Fig. 1X; Figs. 2A to 2D). Areolae on the canal raphe of the same size and shape as those of the valve face. Each row of transapical striae corresponds to one rounded or elongate areola on canal raphe (Fig. 1X). A row of areola present on the canal raphe separated from those on the valve face with a distinct apically oriented series of vimines which correspond to the place on the valve interior where the fibulae are sealed off (Figs. 2A to 2D).

Valve face internally flat with perpendicular valve mantle and a single row of areolae above the fibulae. Raphe slit without a break (*i.e*. central nodule) while distal raphe endings terminate in distinct helictoglossae (Figs. 2A to 2D). Raphe slit enclosed by the canal raphe and subtended by fibulae. Fibulae irregularly distributed along the valve length (Figs. 2A to 2D). The spaces between fibulae variable and no obvious tendency is recognizable. Fibulae small, narrow and similar in shape, 20–22 in 10 μm (Figs. 2A to 2D). Each fibula is borne from two virgae. Striae-forming areolae positioned in shallow depressions, evenly spaced. Areolae circular, and occluded by hymenes (Figs. 2A to 2D), ca. 55 in 10 μm.

*Similar taxa: Nitzschia anatoliensis* sp. nov. shows some degree of resemblance to several species including *Nitzschia aurariae* Cholnoky 1966, *Nitzschia communis* Rabenhorst 1860, *Nitzschia imae* Álvarez-Blanco & S. Blanco 2013, *Nitzschia ovalis* H.J.Arnott 1880 and *Nitzschia pusilla* Grunow 1862.

*Differential diagnosis: Nitzschia anatoliensis* is morphologically extremely similar to *N. aurariae*; both taxa have similar linear-elliptic valve outline, slightly parallel valve margins with broadly rounded apices (Table 1). However, our data (based on one clone) suggests that *N. anatoliensis* may be distinguishable from *N. aurariae* on the basis of fibula density: according to Krammer & Lange-Bertalot (1988), *N. aurariae* has higher fibula density (15–18 in 10 µm). Furthermore, *N. anatoliensis* resembles *N. imae* in terms of valve outline; however, *N. imae* is much wider (5.9–6.3 µm) with lower stria and fibula densities and its ends are slightly protracted (Table 1). *N. anatoliensis* can be also compared with *N. communis*; however, the latter species is also wider (4.0–5.8 µm) and has coarser striae and fibulae. *N. pusilla* is another similar taxon but it has linear-lanceolate or linear valve outlines with slightly protracted ends. Finally *N. ovalis* is also similar but has an more elliptic valve outline with lightly protracted apices, is wider (4.5–6.6 µm) and has a lower fibula density (12–16 in 10 µm) (Table 1).

**Table 1:** Comparison of morphometric data and morphological characteristics of *N. anatoliensis* and morphologically similar *Nitzschia* species.

	*Nitzschia anatoliensis* sp. nov.	*Nitzschia aurariae* Cholnoky	*Nitzschia communis* Rabenhorst	*Nitzschia* *imae* Álvarez-Blanco, I. & Blanco, S.	*Nitzschia* *ovalis* H.J. Arnott	*Nitzschia* *pusilla* Grunow
Source of data	This article *N* = 54	Krammer & Lange-Bertalot, 1988	Krammer & Lange-Bertalot, 1988	Álvarez-Blanco & Blanco, 2013	Krammer & Lange-Bertalot, 1988	Krammer & Lange-Bertalot, 1988
Valve shape	Linear-elliptic	Linear-elliptic	Elliptic, linear-elliptic to linear	Linear-elliptic to linear	Elliptic to linear-elliptic	Linear-lanceolate to linear
Central nodule	Absent	Absent	Absent	Absent	Absent	Absent
Apex shape	Broadly rounded, not protracted	Broadly rounded, not protracted	Broadly rounded, slightly protracted	Broadly rounded, slightly protracted	Broadly rounded, slightly protracted	Broadly rounded, slightly protracted
Valve length (µm)	7.8–16.1	6.5–18.0	6.0–40.0	16.5–26.0	13.0–22.5	8.0–33.0
Valve width (µm)	2.7–3.8	2.5–4.0	4.0–5.8	5.9–6.3	4.5–6.6	2.5–5.0
Striae in 10 µm	48–52	46–53	28–38	40–45	ca. 42	(40) 43–55
Fibulae in 10 µm	20–22	(13) 15–18	(8) 10–14	15–17	12–16	14–20 (24)

**Genomic and phylogenetic analyses**

**Figure 3:**
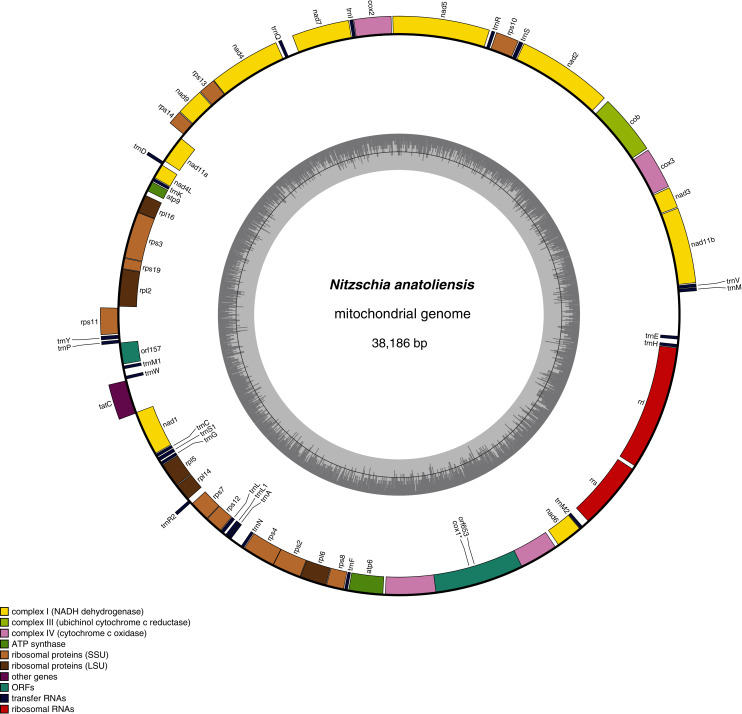
Genomic map of the mitochondrial genome of *N. anatoliensis*.

The mitogenome of *Nitzschia anatoliensis* is 38186-bp long (Fig. 3). It is registered on GenBank with accession number MT742552. It contains a total of 61 genes, encoding 35 proteins, two rRNAs and 24 tRNAs. A conserved open reading frame (*orf157*) was detected within the synthenic bloc *rps11*–*orf157—tatC* described by Pogoda et al. (2019). The *cox1* gene contains a group II intron that encodes a putative reverse transcriptase. Genes are encoded on both DNA strands. The sequence of the mitogenome is available as Supplemental File 1.

**Figure 4:**
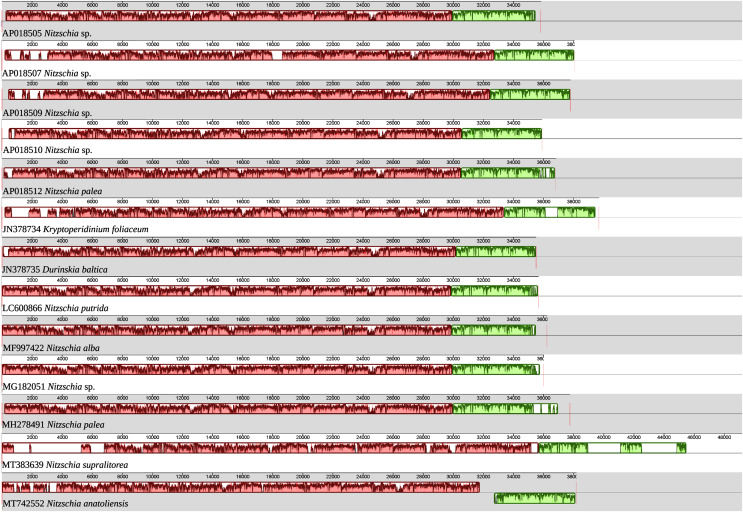
MAUVE alignment of the mitochondrial genome of *N. anatoliensis* with other species of *Nitzschia* spp. and dinotoms. The coloured boxes indicate the blocks of synteny. The first block of synteny (in red) is 30 Kb to 35 Kb long. It is followed by a second block (in green) located on the negative-sense strand in *Nitzschia anatoliensis*.

As illustrated by the MAUVE alignment (Fig. 4), the mitogenome of *N. anatoliensis* singularizes itself from other *Nitzschia* spp. and the two dinotoms. The cluster of genes containing *trnE*, *trnH*, *rrl*, *rrs*, *trnM*, *nad6* is located on the opposite strand compared to these species. The overall size of the genome is similar with the other species, except for the case of *Nitzschia supralitorea* Lange-Bertalot 1979 whose mitogenome is 49,250-bp long (see Gastineau et al., 2021a).

**Figure 5:**
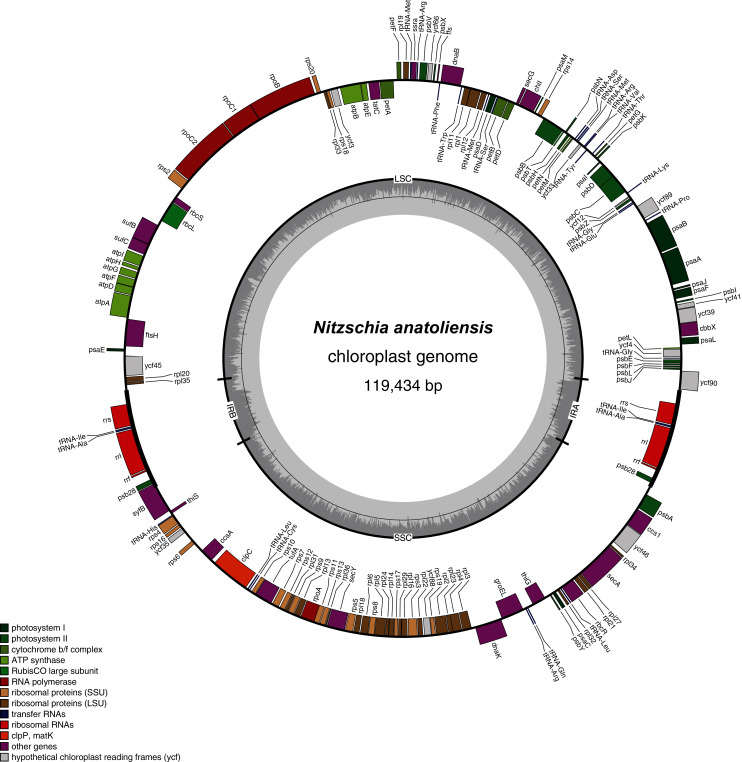
Genomic map of the plastid genome of *N. anatoliensis*.

The plastid genome is 119,434-bp long (Fig. 5). It is registered on GenBank with accession number MT742551. It displays the usual quadripartite organization, with two identical inverted repeats of 6,948 bp, a large single-copy (LSC) of 64,054 bp, and a small single-copy (SSC) of 41,484 bp. Each inverted repeat contains three rRNA genes (*rrf*, *rrs* and *rrl*), two tRNA genes (*trnI* and *trnA*), and the protein-coding gene *psb28* as well as the partial coding sequence of *syfB*. The LSC harbors 75 protein-coding genes and 18 tRNA genes, while the SSC contains 52 protein-coding genes and 6 tRNA genes. No large non-conserved ORF was identified, to be compared for example with *Seminavis robusta* D.B. Danielidis & D.G. Mann (Brembu et al. 2014) or with Haslea silbo Gastineau, Hansen and Mouget (Gastineau et al. 2021b). Genes are encoded on both strands. Total length is similar to the two available plastid genomes of the genus *Nitzschia*, obtained from *Nitzschia palea* (Kützing) W. Smith AP018511 (119,116 bp long) and *Nitzschia palea* (Kützing) W. Smith 1856 MH113811 (119,449 bp long). The genome of *N. palea* contains a 449 amino acid large ORF not detected in *N. anatoliensis*, and its inverted repeats have a different organization, lacking the *psb28* gene but containing the hypothetical conserved protein *ycf89* instead. The sequence of the plastid genome is available as Supplemental File 2.

**Figure 6:**
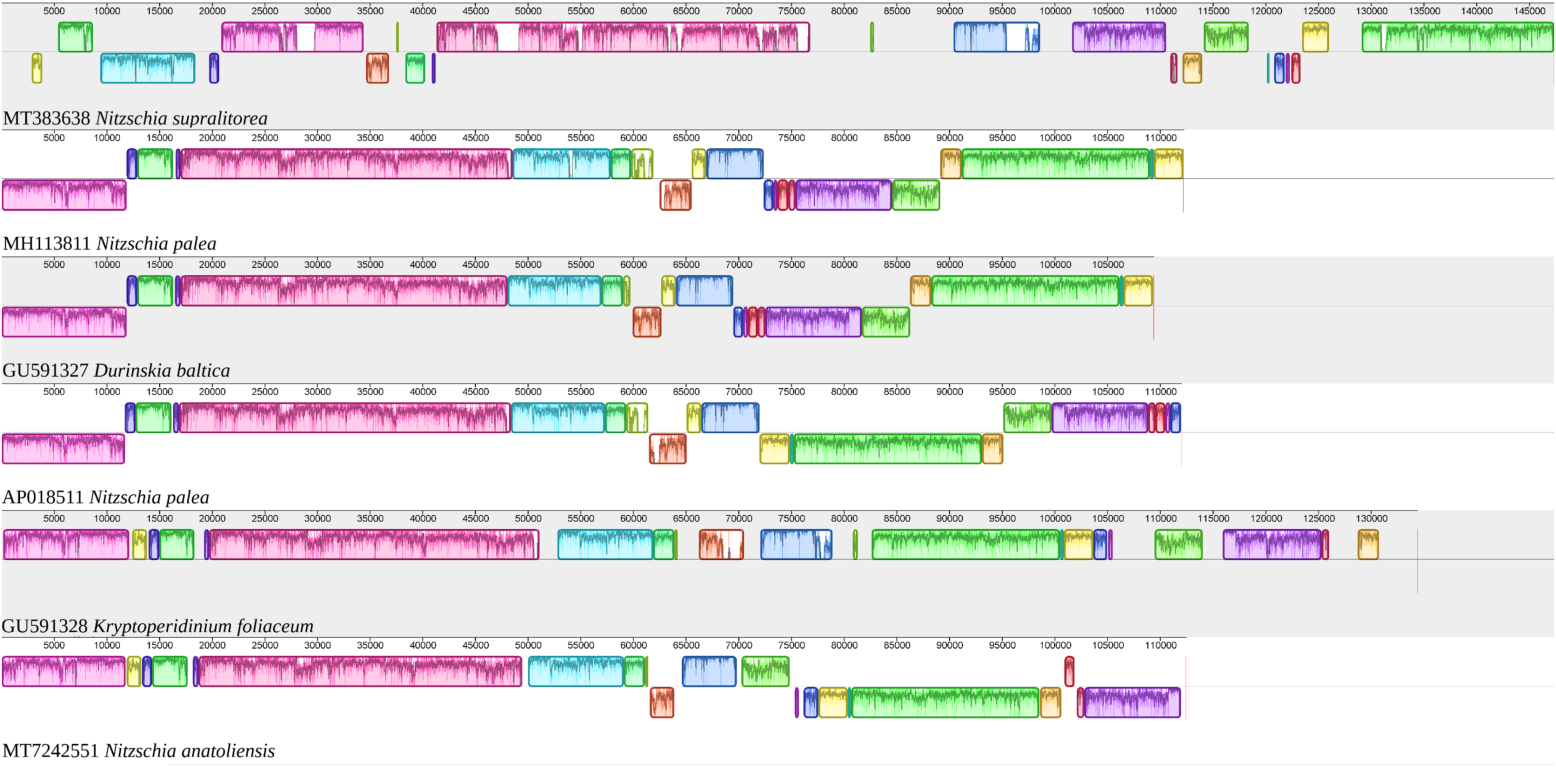
MAUVE alignment of the plastid genome of *N. anatoliensis* with other species of *Nitzschia* spp. and dinotoms. The coloured boxes indicate the blocks of synteny.

The MAUVE alignment (Fig. 6) illustrates the conservation of the LSCs between *N. anatoliensis* and the endosymbiont of *K. foliaceum*. The gene order in the LSC is identical, except for a small cluster of three genes (*rpl35*-*rpl20*-*ycf45*) near the IR, and which is on different strands. The SSC is more rearranged, and it is also worth noting that compared to *K. foliaceum*, *N. anatoliensis* cpDNA does not display non-conserved ORFs or putative *serC* and *xerC* genes originating from plasmids (Imanian, Pombert & Keeling, 2010). *Nitzschia supralitorea* has the most distinct plastid genome compared to the other, both in terms of size and gene order.

A 8,686 bp fragment containing all nuclear ribosomal genes (18S-ITS1-5.8S-ITS2-28S) was also recovered and deposited on GenBank as MT740317. The megablast analysis of *SSU* gene displays 100% identity with those of two diatoms referenced as *Nitzschia* cf. *communis* (KM387718 and KM387719). Also, there was 99.86% identity with a diatom described as *N. communis* (KM387717). However, two other sequences registered as *N. communis* (AJ867014 and AJ867278) showed a 98.69% identity, a value lower than those obtained for a strain of *N. pusilla* (KY320390) or the species *Nitzschia bizertensis* B.Smida, N. Lundholm, A. S. Hlaili & H. H. Mabrouk (KF955285) (Bouchouicha Smida et al., 2014). For the *LSU* gene, the best match was *Nitzschia palea* (HF679202) with 93.25% identity. *N. communis* (AF417661) came only as the 10^th^ match, with a 92.97% identity. The sequence of the cluster of nuclear ribosomal genes is available as Supplemental File 3.

The best matches for the blastn analyses of *rbcL* were with the endosymbiont of *K. foliaceum* (GU591328 and U31876), with 97.89% and 97.28% sequence identities, respectively. A comparison of trimmed *rbcL* genes from *N. anatoliensis* and similar species showed the following results: for *N. communis* MN696775, 1,185 bp long fragments, 92.24% of identity; for *Nitzschia pusilla* (1,188 bp long fragments), it ranged between 91.33% and 94.28% (HF675109, HF675108, HF675110, KY320329, KY320323, KY799146, MN718763, KY863494, MN696779, KY863493); for two strains of *Nitzschia aurariae* (1,024 bp fragments), identities 90.62% with *N. aurariae* MH898880 and 91.50% with *N. aurariae* KT943663.

**Figure 7:**
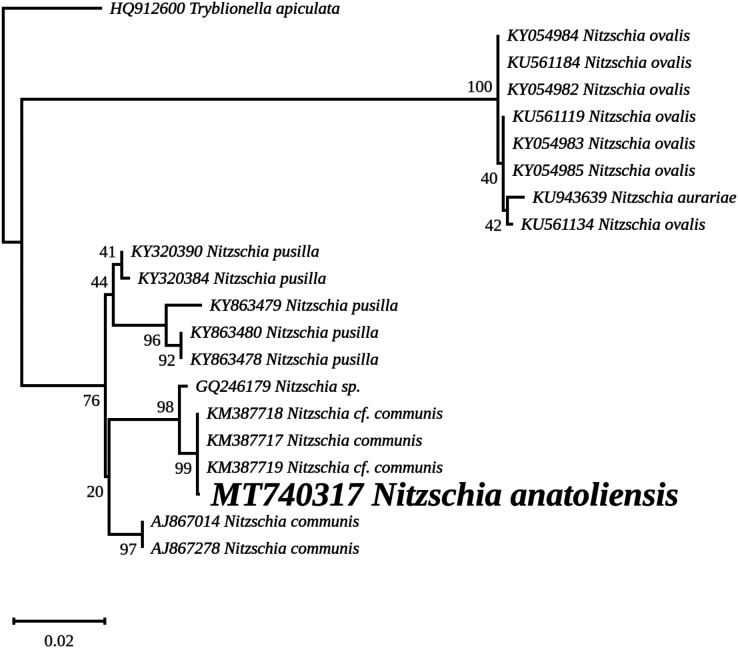
Maximum likelihood phylogeny inferred from an alignment of 21 partial *SSU* genes. The best-scoring RAxML tree (log likelihood = −2,936.023088) is presented. Bootstrap support values are reported on the nodes. Evolutionary analyses were conducted using RAxML version 8, with the secondary structure and the GTR 16-state model and 100 bootstrap replications. The scale indicates the number of substitutions per site.

**Figure 8:**
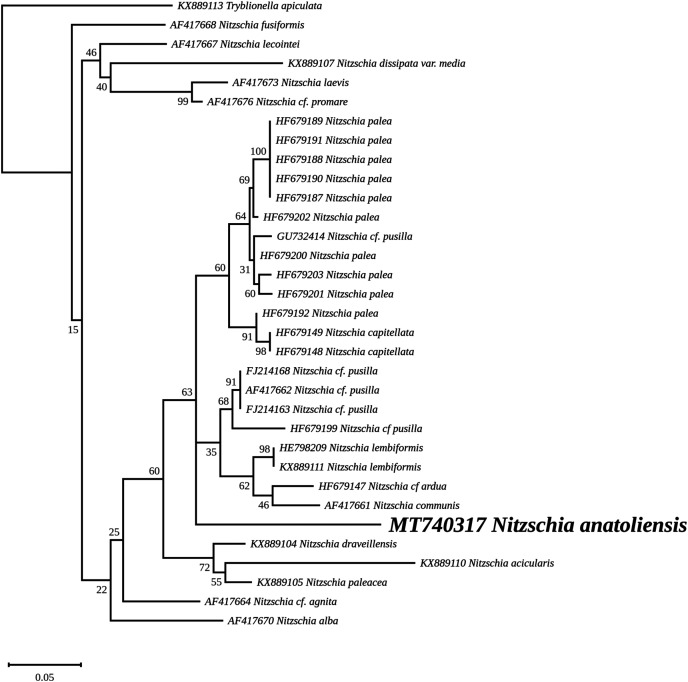
Maximum likelihood phylogeny inferred from an alignment of 34 partial *LSU* genes. The best-scoring RAxML tree (log likelihood = −2,112.349181) is presented. Bootstrap support values are reported on the nodes. Evolutionary analyses were conducted using RAxML version 8, with the secondary structure and the GTR 16-state model and 100 bootstrap replications. The scale indicates the number of substitutions per site.

The nuclear *SSU* phylogeny was not intended to investigate relationships over a broad phylogenetic range; so taxon sampling focused on *Nitzschia* species whose morphologies were compared in the differential diagnosis reported here (Fig. 7). This phylogeny strictly discriminated *N. anatoliensis* from clones identified as *N. ovalis*, *N. aurariae* and *N. pusilla*. The nuclear *LSU* phylogeny also clearly distinguished *N. anatoliensis* from *N. communis* AF417661, and also from *N. pusilla* (Fig. 8).

**Figure 9:**
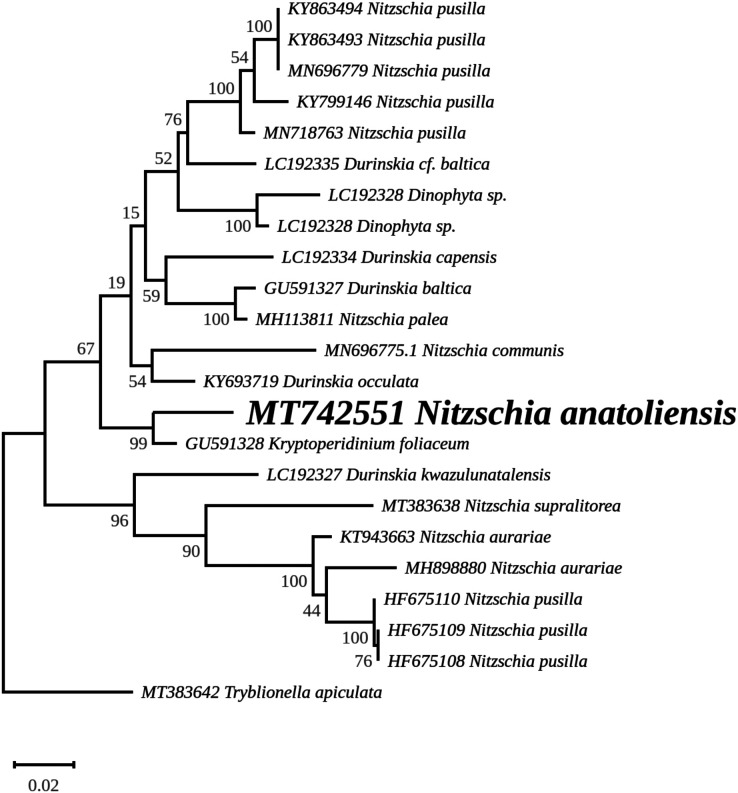
Maximum likelihood phylogeny inferred from an alignment of 23 partial *rbcL* genes from diatoms and dinotoms. The best-scoring RAxML tree (log likelihood = −3,341.42) is presented. Bootstrap support values are reported on the nodes. Evolutionary analyses were conducted using RAxML version 8, with the GTR + I + G model and 1,000 bootstrap replications. The scale indicates the number of substitutions per site.

**Figure 10:**
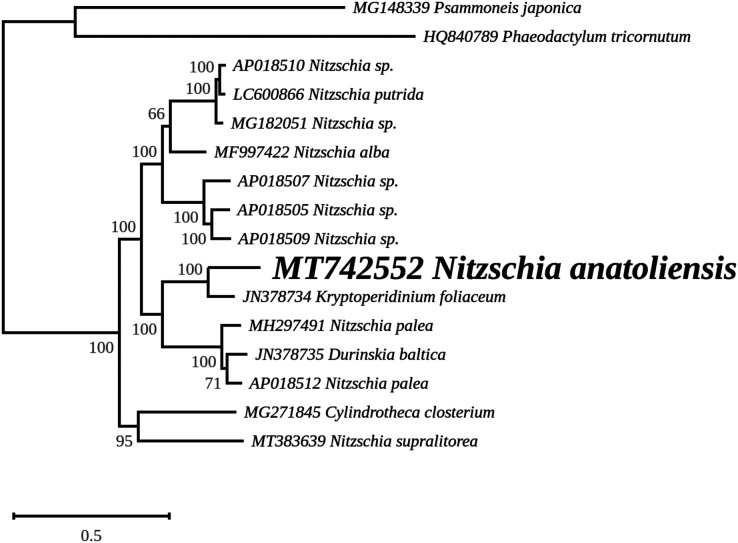
Maximum likelihood phylogeny inferred from an alignment of concatenated protein coding genes from 16 mitochondrial genomes of diatoms and dinotoms. The best-scoring RAxML tree (log likelihood = −398,630.471192) is presented. Bootstrap support values are reported on the nodes. Evolutionary analyses were conducted using RAxML version 8, with the GTR + I + G model and 1,000 bootstrap replications. The scale indicates the number of substitutions per site.

The *rbcL* tree includes sequences from various dinotoms and has been rooted with *Tryblionella apiculata* W. Gregory 1857. It associates *N. anatoliensis* with *K. foliaceum*. While some other node values were low, the tree clearly distinguished between *N. anatoliensis* and some of the morphologically similar species such as *N. aurariae* or *N. pusilla*, as well as it also clearly discriminates it from *N. communis* (Fig. 9). The trees inferred from concatenated mitochondrial genes (Fig. 10) unambiguously associated *N. anatoliensis* with the dinotom *K. foliaceum*, this clade being associated with another one containing *N. palea* and *D. baltica*, in both cases, with very strong bootstrap values. Surprisingly, *N. supralitorea* appears closer to *Cylindrotheca closterium* (Ehrenberg) Reimann & J. C. Lewin 1964, that we expected to appear with the two other outgroup species.

## Discussion

Based on morphological comparisons with similar species, but above all the use of several molecular markers, it is clear that the strain from Van Lake is a new species, *Nitzschia anatoliensis*. It originates from the benthos of an extreme environment, with unusually high sodium bicarbonate concentration resulting in high pH. It is among the few diatom species able to live in the waters of Van Lake (Gessner, 1957).

The ML phylogeny inferred from the nuclear *SSU* gene recovered *N. anatoliensis* with three strains of *N. communis* (KM387717, KM387718 and KM387719) but discriminated it from two others (AJ867014 and AJ867278) yet with low bootstrap values. In comparison, the *LSU* based phylogeny was more efficient in discriminating *N. anatoliensis* from *N. communis* strain M1762 (AF417661) from the Cologne Botanical Garden (Germany). The three strains belonging to the same clade as *N. anatoliensis* were either labeled as *N. communis* or *Nitzschia* cf. *communis*, and all share similar origins: they were all isolated from Siberian soda lakes (with a pH amounting to ca. 10; Samylina et al., 2014; Sapozhnikov et al., 2016). However, a weak point of the referenced publications was the lack of SEM documentation in support of the taxonomic identification. On the other hand, the strains labelled as *N. communis* NCOM1 (AJ867014) and *N. communis* FDCC L408 (AJ867278) originated from Luxembourg and Arizona, respectively (the second strain being now registered as UTEX LB FD58) and they do not seem to come from alkaline environments. The separation of *N. communis* into two clades based on the nuclear *SSU* gene also appears in the work of Samylina et al. (2014) and in Yamada, Sym & Horiguchi (2017). In terms of the molecular clades distinguished very recently by Mann et al. (2021), *N. anatoliensis* and *N. communis* would both belong to clade 6B.

Therefore, we propose that the *N. communis* strains described by Samylina et al. (2014) and Sapozhnikov et al. (2016), clustering in the same clade as the *Nitzschia* species we examined here and with a 100% identity of their *SSU* partial genes, are in fact *N. anatoliensis* sp. nov. This species can be found at very distant locations, the Van Lake and some alkaline lakes of the Kulunda Steppes, which are approximately 3,200 km apart. But its exact geographical distribution is yet unknown and remains a question that might be addressed. Whether or not this species is restricted to alkaline environment is an interesting issue that warrants investigation using the same molecular method that led to the description of *N. anatoliensis*. A remaining question is whether or not *N. communis* identified by Legler & Krasske (1940) and mentioned by Gessner (1957) is conspecific with *N. anatoliensis*. One way to answer this question could be to study remaining slides from the Krasske collection, curated in Kassel (Germany).

We should also emphasize that in addition to its previous confusion with *N. communis*, *N. anatoliensis* could have also been overlooked because of its strong similarity with *N. aurariae*. With regards to this challenge, molecular barcoding has been a crucial tool to discriminate between these two species, which belong to distant clusters.

An unexpected outcome of our study is that *N. anatoliensis* appeared as a sister group to *K. foliaceum*, a cosmopolitan species of dinotom (Figueroa et al., 2009; Saburova, Polikarpov & Al-Yamani, 2012; Lewis et al., 2018). Dinotoms are dinoflagellates that underwent a third endosymbiosis event during which they acquired their mitogenome and plastid genome from a diatom (Imanian, Pombert & Keeling, 2010; Imanian et al., 2012; Hehenberger et al., 2014; Crowell, Nienow & Cahoon, 2019; Yamada et al., 2019). This event is different from the endosymbiosis event that led to the reduced chloroplast-related minicircles found in most photosynthetic dinoflagellates (Howe, Nisbet & Barbrook, 2008). Our results raise questions concerning the nature of the common ancestor of *N. anatoliensis* and *K. foliaceum*’s endosymbiont. We suggest that sequencing more organellar genomes of delicate, finely striate *Nitzschia* is needed to confirm/extend these results. Such a program of extended seqencing may also lead to the discovery of cryptic species, in a similar way to the process that led to the description of *N. anatoliensis*.

## Supplemental Information

10.7717/peerj.12220/supp-1 Supplemental Information 1 Fasta sequence of the mitochondrial genome.

10.7717/peerj.12220/supp-2 Supplemental Information 2 Fasta sequence of the chloroplast genome.

10.7717/peerj.12220/supp-3 Supplemental Information 3 Fasta seqeunce of the cluster of nuclear ribosomal genes.

10.7717/peerj.12220/supp-4 Supplemental Information 4 Measurements in LM and SEM of Nitzschia anatoliensis. The lengths and widths measured on LM and SEM pictures and the striae and fibulae measured on SEM pictures.

## Additional Information and Declarations

### Data Availability

The following information was supplied regarding data availability: The sequences are available in NCBI (mitogenome, MT742552; chloroplast genome, MT742551; rRNA genes, MT740317) and in the Supplemental Files. The measurements performed on light and scanning electron microscopy are in the Supplemental File.

### New Species Registration

The following information was supplied regarding the registration of a newly described species: Nitzschia anatoliensis sp. nov. (diatoms-algae).
